# Long-term outcomes of JIA-associated uveitis: a systematic review and meta-analysis

**DOI:** 10.1136/rmdopen-2025-006071

**Published:** 2025-11-27

**Authors:** Laura Scagnellato, Giacomo Cozzi, Mariagrazia Lorenzin, Gianluca Poncina, Stefano Rizzetto, Roberta Ramonda

**Affiliations:** 1 Rheumatology Unit, Department of Medicine—DIMED, Padova University Hospital, Padova, Italy

**Keywords:** Arthritis, Juvenile, Uveitis, Anterior, Outcome Assessment, Health Care, Biological Therapy

## Abstract

**Background and aim:**

Juvenile idiopathic arthritis-associated uveitis (JIAU) is a potentially blinding condition beginning in childhood. Flare-ups may occur throughout life, leading to cumulative ocular damage. This systematic review aimed to summarise the latest evidence on long-term ocular damage in adults with JIAU, focusing on studies published in the past 20 years.

**Methods:**

The review protocol was registered in PROSPERO (ID 1033522). We included human studies on JIAU with at least 5 years of follow-up and/or young people ≥18 years old, published after 2000. Study quality was assessed using the Newcastle-Ottawa Scale. We extracted the cumulative incidence of complications and performed a meta-analysis to obtain pooled estimates (PE), assess heterogeneity, sensitivity, robustness and reporting bias using R packages.

**Results:**

22 retrospective or cohort studies were eligible, encompassing 2208 long-term JIAU cases. Study quality was rated as good in 10, fair in 7 and poor in 5. Despite high heterogeneity (p<0.01), sensitivity analyses supported robustness. Evidence of reporting bias was found for severe visual impairment and cataract. The pooled incidence of severe visual impairment was 16.6% (95% CI 9.9% to 22.6%) at approximately 18 years. Cataract was the most frequent complication (PE 44.3% (95% CI 27.5% to 61.7%)), followed by glaucoma, synechiae, band keratopathy and hypotony. In cohorts with >50% biologic exposure, PE were numerically lower.

**Conclusion:**

Long-term prevalence and cumulative incidence of JIAU complications may persist in adulthood, with >10% developing severe visual impairment despite biologic therapy.

**WHAT IS ALREADY KNOWN ON THIS TOPIC**

- Juvenile idiopathic arthritis-associated uveitis causes long-term ocular damage despite the advent of biologic therapy.

**WHAT THIS STUDY ADDS**

- Even in the biologics era, over 10% of young people may develop severe visual impairment.

**HOW THIS STUDY MIGHT AFFECT RESEARCH, PRACTICE OR POLICY**

- Highlights the need for improved therapies and a clearer understanding of disease mechanisms.

## Introduction

 Juvenile idiopathic arthritis (JIA)-associated uveitis (JIAU) is one of the most frequent forms of anterior uveitis among children and is a potentially blinding condition.

The prevalence of JIAU ranges from 11.6% to 30% among children with arthritis.^1 2^ Several risk factors associated with the development of uveitis in children or young people (YP) with JIA have been identified, including female sex, oligoarticular subtype and antinuclear antibodies positivity, which are all markers of risk stratification in clinical practice.^1 3^ Screening programmes for children with JIA are considered standard of care, and their implementation has been consolidated in paediatric care.^4^

JIAU is typically chronic, asymptomatic, anterior and non-granulomatous, whereas acute anterior uveitis is typically associated with enthesitis-related arthritis and Human Leukocyte Antigen (HLA)-B*27 positivity and presents with clinically overt symptoms, like redness, photophobia, eye pain and/or blurred vision. Due to its asymptomatic presentation, chronic anterior uveitis is often diagnosed when serious visually-impairing complications have already occurred, unless children are screened early. Cystoid macular oedema is a severe manifestation of chronic anterior uveitis that may occur if undiagnosed and untreated in patients affected by JIA. Complications of JIAU include cataract, glaucoma, posterior synechiae and band keratopathy, which may result in rapid visual impairment and/or ocular hypotony if untreated.^5^ In a previous systematic review comprising 26 eligible series, the cumulative incidence of adverse outcome (visual acuity <20/40 bilaterally) was 9.2%; cataract 20.5%; glaucoma 18.9% and band keratopathy 15.7%.^6^ The management of JIAU involves the use of both topical and systemic agents, such as glucocorticoids, conventional synthetic disease-modifying antirheumatic drugs (csDMARDs), such as methotrexate, and biologic agents (eg, inhibitors of tumour necrosis factor (TNFi) and interleukin 6 (IL-6i)).^3 7^

## Objectives

The primary outcome of our study was to evaluate severe uveitis complications in YP with a diagnosis of JIAU via a systematic review of the existing literature. The secondary outcomes were to assess the persistence of active eye disease and/or cystoid macular oedema, and each individual complication (ie, cataract, secondary glaucoma, band keratopathy, posterior synechiae, papilloedema and ocular hypotony).

## Methods

### Systematic review

#### Study selection

We conducted a search in PubMed and Embase on 15 February 2025 using index terms “juvenile idiopathic arthritis”, “uveitis”, “cataract”, “glaucoma”, “band keratopathy” and “blindness” (full strings reported in online supplemental material). The study protocol was registered in PROSPERO with the ID number 1 033 522 and designed according to the Preferred Reporting Items for Systematic Reviews and Meta-Analyses guidelines. This study did not involve human participants and did not require approval by an Ethics Committee(s) or Institutional Board(s). Patients and the public were not involved in the design and conduct of the study.

The initial inclusion criteria for the study were: (a) all study types reporting on more than 10 JIAU YPs’ manifestations and complications, including persistently active disease, cystoid macular oedema, cataract, band keratopathy, ocular hypertension, posterior synechiae, ocular hypotony, papilloedema and epiretinal membrane formation; (b) JIAU median disease follow-up of at least 5 years. Reports on systemic JIA, reports not in the English language or reports whose full text was not available, as well as studies included in a previous review published in 2006, were excluded.^6^ A second search on PUBMED conducted on 28 August 2025 was integrated and crosschecked with the first one.

**Figure 1:**
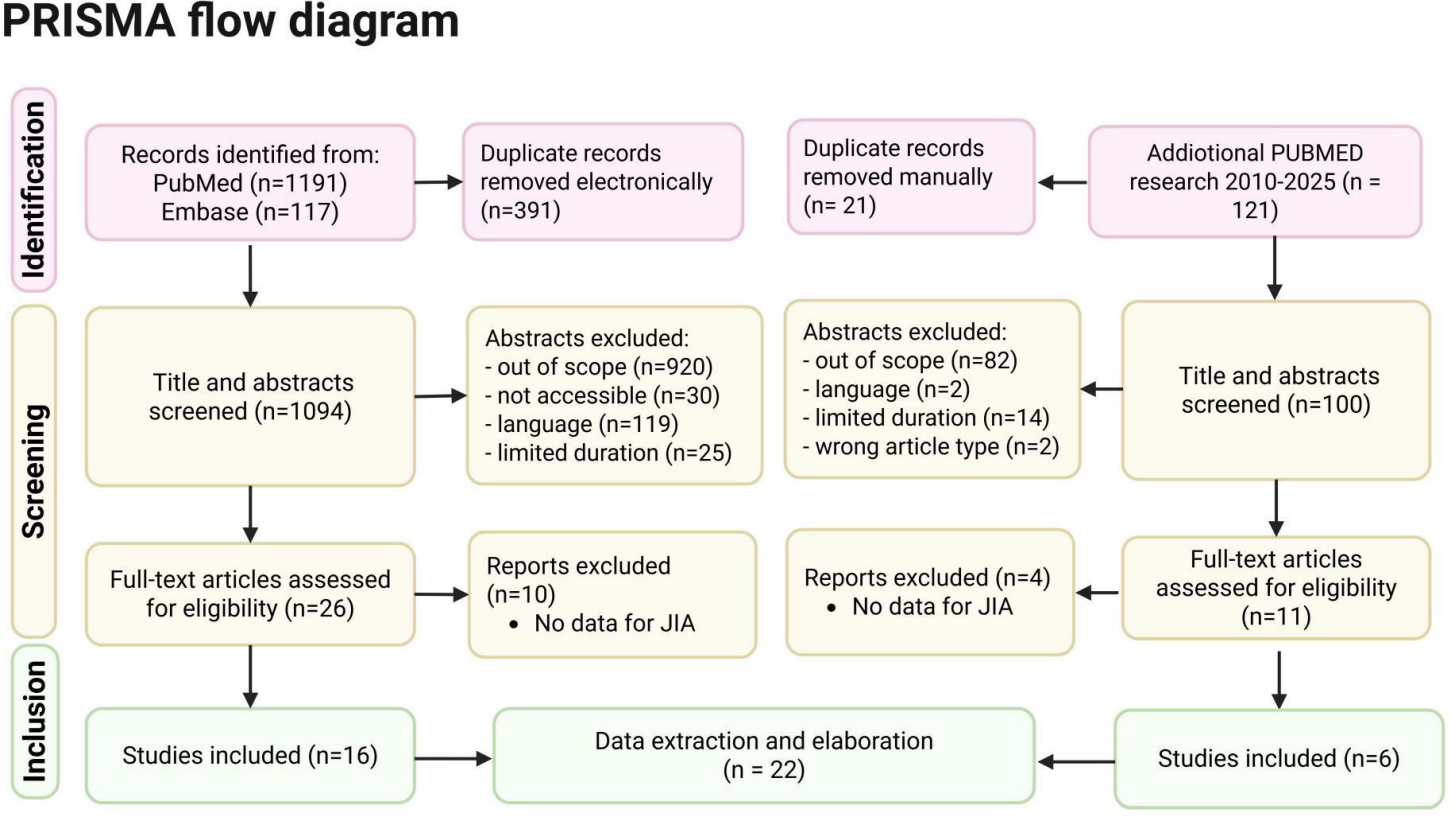
Flow diagram of the current systematic research retrieving 22 significant studies. PRISMA, Preferred Reporting Items for Systematic Reviews and Meta-Analyses.

The initial search yielded 1308 single entries, which were narrowed down to 1094 after duplicate electronic detection and assisted removal using the Rayyan platform.^8^ 26 eligible studies were selected by two independent reviewers (LS and Matteo Molfese, MM) who examined titles and abstracts (figure 1). After further full-text analysis, only 16 papers were included. The second research on PUBMED yielded 121 results that were ultimately narrowed down to six eligible studies, adding up to 22 studies for the final analysis. A third independent reviewer (RR) acted as a tiebreaker to resolve any disagreement.

#### Data extraction

Each report was identified by author name, year of publication, full title and study design (retrospective versus prospective). For each study, we recorded in an electronic datasheet the following: the number of YP affected by JIAU, each complication, the median follow-up time (years) and related treatments. The number of affected eyes was also reported when appropriate. Severe visual impairment was defined as visual acuity ≤1 in Logarithm of the Minimum Angle of Resolution (LogMAR) scale or equivalent in Snellen (≤20/200), metric (≤ 6/60) or decimal units (≤ 0.1). Given the high heterogeneity of the studies, the end-period cumulative incidence of each complication was preferred; when this information was not available, we retrieved the prevalence at the last possible follow-up time point. With regard to active uveitis, the prevalence at the last follow-up was recorded as a percentage. Missing data were researched in online supplemental material, when available.

### Quality assessment

Retrieved studies were all cohort studies; we identified no randomised controlled trial or controlled trial pertinent to our research question and meeting our inclusion criteria. All 22 studies were scored by two independent reviewers (LS and MM) using the Newcastle-Ottawa Scale for cohort studies, according to the user manual. Any conflict was resolved through discussion with the other authors. The main factors considered for quality assessment were the design of the study (retrospective for most) and the number of centres involved for the selection domain, the description of JIA cases and the detailed description of therapeutic protocol for comparability, and the description of detailed outcomes for all cases in the outcome domain.

### Synthesised results and meta-analysis

All analyses and figures were obtained with R V.2024.12.1 using the meta, metafor and ggplot2 packages. RData files will be available on direct request to the authors.

Pooled estimates (PE) for each long-term outcome were obtained by fitting a random effects model based on median cumulative incidence and YP numerosity from each study with comparable proportions (percentage of YP affected by a specific outcome at a given time point >5 years after uveitis onset). Studies that did not report comparable estimates were not included in the meta-analysis. Heterogeneity was assessed using the meta package tools for each outcome individually. Sensitivity analyses were performed using the leave-one-out method and the cumulative meta-analysis function of the metafor package. Publication bias was assessed using funnel plots and Egger’s test for asymmetry. Considering the significant impact of TNFi and other biologics on the outcomes of JIAU, we initially planned to stratify the analysis based on the recruitment period. However, no studies provided sufficient data to compare outcomes between the prebiologic era (before 2010) and the biologic era (after 2010). Because of substantial heterogeneity in how recruitment periods were reported (some studies used date of referral, others disease onset) and because biologics were often introduced during follow-up, a reliable ‘era-based’ classification (prebiologic versus postbiologic availability) was not feasible. Therefore, we prespecified our main comparison according to the percentage of biologic exposure at the study level: high exposure (≥50% of patients treated with biologic DMARDs (bDMARDs) at any time) versus low exposure (<50%). This approach aimed to mitigate misclassification due to off-label use or delayed treatment initiation and to better reflect the actual therapeutic intensity of each cohort. Moderator analyses considered: (1) bDMARD exposure proportion (≥50% vs <50%), (2) follow-up duration (>15 years and ≥15 years) and (3) year of publication (before or after 2020).

## Results

### Included studies and populations

From the 1308 references retrieved initially, our systematic review ultimately included 22 single studies on the long-term outcomes of JIAU, describing disease activity and complications. All studies were retrospective or cohort studies and published after 2000 (table 1). All studies reported were from centres in Europe (16), the USA (5) and Australia (1); no studies from other continents were found. 10 studies were deemed good quality, seven studies fair quality and five studies poor. Poor quality was assigned mainly due to the lack of comparability of the presented cases (table 2). The studies that presented the highest scores were those where the use of bDMARDs was described thoroughly.

**Table 1:** Summary of included studies

First author	Year of publication	Number of JIA YP (number of eyes)	Median follow-up (years)	Outcomes considered	Treatments
Kotaniemi^49^	2001	14	11	Severe visual loss	/
Camuglia^18^	2009	17 (30)	17.3	Ocular hypotony, synechiae, cataract	GC, csDMARDs, surgery
Marvillet^9^	2009	69 (118)	6.8	Severe visual loss, glaucoma, cataract, synechiae, band keratopathy	GC, csDMARDs, bDMARDs (<50%), surgery
Tu^16^	2014	20 (37)	25.6	Active uveitis, glaucoma, cataract	bDMARDs, surgery
Oray^19^	2016	77 (135)	12.3	Active uveitis, CME, severe visual loss, glaucoma, cataract, synechiae, band keratopathy, hypotony	csDMARDs, bDMARDs (>50%), surgery
Stroh^50^	2016	108 (196)	30	Active uveitis, severe visual loss, cataract, synechiae, band keratopathy	GC, surgery
Dimopoulou^51^	2017	11	17	Severe visual loss	/
Kolomeyer^17^	2017	19 (33)	25.6	Active uveitis, CME, severe visual loss, glaucoma, cataract, synechiae, band keratopathy, hypotony	GC, csDMARDs, bDMARDs (>50%), surgery
Ferrara^14^	2017	100 (190)	5.35	CME, severe visual loss, glaucoma, cataract, synechiae, band keratopathy, hypotony, papilloedema	GC, csDMARDs, surgery
Cann^13^	2018	19 (34)	10.0	CME, severe visual loss, glaucoma, cataract, synechiae, band keratopathy, hypotony	GC, csDMARDs, bDMARDs, surgery
Kulik^15^	2019	24 (34)	10.9	CME, severe visual loss, glaucoma, cataract, synechiae, band keratopathy, hypotony	Surgery
Morelle^52^	2021	91 (161)	5.2	CME, severe visual loss, glaucoma, cataract, synechiae, band keratopathy, papilloedema	GC, csDMARDs, bDMARDs (<50%), surgery
Rypdal^12^	2020	96	18	Active uveitis, CME, severe visual loss, glaucoma, cataract, synechiae, band keratopathy, hypotony	csDMARDs, bDMARDs (>50%), surgery
Leinonen^11^	2020	20 (26)	12.3	Active uveitis, CME, severe visual loss, glaucoma, cataract, synechiae, band keratopathy, hypotony	GC, csDMARDs, bDMARDs (<50%), surgery
Marelli^53^	2021	125	9.2	CME, severe visual loss, glaucoma, cataract, synechiae, band keratopathy	GC, csDMARDs, bDMARDs (>50%), surgery
Skarin^54^	2022	55	40.7	Active uveitis, severe visual loss, glaucoma, cataract	/
Markomichelakis^10^	2023	80	10	CME, severe visual loss, glaucoma, cataract, synechiae, band keratopathy, hypotony, papilloedema	csDMARDs, bDMARDs (<50%), surgery
Iannone^55^	2023	60	12.5	/	csDMARDs, bDMARDs (<50%), surgery
Huard^21^	2023	27	5	Severe visual loss, glaucoma	GC, csDMARDs, bDMARDs (>50%), surgery
Akhavanrezayat^56^	2024	54	9.5	Active uveitis, glaucoma, cataract, band keratopathy	/
Mesa-Del-Castillo^33^	2024	263	6.2	CME, severe visual loss, glaucoma, cataract, hypotony, papilloedema	GC, csDMARDs, bDMARDs (>50%), surgery
Siiskonen^32^	2025	88 (143)	8.45	Cystoid macular oedema, severe visual loss, glaucoma, cataract, hypotony, papilloedema	Surgery

bDMARDs, biological disease-modifying antirheumatic drugs; CME, cystoid macular oedema; csDMARDs, conventional synthetic disease-modifying antirheumatic drugs; GC, systemic glucocorticoid use; JIA, juvenile idiopathic arthritis; YP, young people.

**Table 2:** Quality assessment of studies included in the review according to the Newcastle-Ottawa Scale, scored by two independent reviewers

First author	Selection	Comparability	Outcome	Points	Quality*
Kotaniemi	3	0	1	4	Poor
Camuglia	2	1	3	6	Fair
Marvillet	2	1	3	6	Fair
Tu	1	0	1	2	Poor
Oray	2	1	2	5	Fair
Stroh	3	1	2	6	Good
Dimopoulou	2	1	2	5	Fair
Kolomeyer	2	1	2	5	Fair
Ferrara	3	0	2	5	Fair
Cann	4	1	2	7	Good
Kulik	2	0	2	4	Poor
Morelle	3	1	2	6	Good
Rypdal	3	2	2	7	Good
Leinonen	2	0	2	4	Poor
Marelli	3	1	3	7	Good
Skarin	3	1	3	7	Good
Markomichelakis	2	1	3	6	Fair
Iannone	2	1	2	5	Fair
Huard	3	2	3	8	Good
Akhavanrezayat	2	1	2	5	Fair
Mesa-Del-Castillo	4	1	3	8	Good
Siiskonen	3	0	2	5	Fair

Studies are arranged in chronological order of publication.

* Poor quality: 0 or 1 star in selection domain OR 0 stars in comparability domain OR 0 or 1 stars in outcome/exposure domain. Fair quality: 2 stars in selection domain AND 1 or 2 stars in comparability domain AND 2 or 3 stars in outcome/exposure domain. Good quality: 3 or 4 stars in selection domain AND 1 or 2 stars in comparability domain AND 2 or 3 stars in outcome/exposure domain.

The selected studies comprised a total of 2578 YP with JIA and 1942 YP with JIAU (table 1). The median follow-up time was 12.25 years (mean 14.7 years, range 5–40.7 years). Data regarding articular disease and serological characteristics were reported in more than 50% of studies. Similarly, most studies reported detailed information on the use of topical and systemic glucocorticoids (50%), csDMARDs (68.2%) and bDMARDs (63.6%). Among reported cases, at least one course was prescribed of either csDMARD (methotrexate) in 720/884 cases (81.4%) or bDMARDs in 683/1036 cases (65.9%) (table 1). The use of bDMARDs was reported in 19%,^9^ 35.7%,^10^ 38.5%^11^ and 54.2%^12^ of cases enrolled before 2012, respectively. Studies with more recent recruitment periods (after 2012) included 755 YP and reported bDMARD exposure in more than 60% of cases, except for the study by Morelle. In addition, Cann and colleagues^13^ reported biologics use in less than 50% of YP with JIA within 10 years of uveitis diagnosis, and in only 30% of those with idiopathic uveitis.

17 studies reported the surgical procedures that JIAU YP underwent during follow-up. In the study by Cann and colleagues^13^ and the study by Ferrara *et al*,^14^ the most common procedure was cataract removal (18 events/639 eye-years of observation, EY and 22% in anterior uveitis YP, respectively), followed by trabeculectomy (11/682 EY) in the study by Cann, and pars plana vitrectomy in the study by Ferrara *et al* (18.1% in all anterior uveitis YP).^13^ Some eyes described in these studies required multiple procedures. In many other included studies, surgical procedures were usually followed by other complications. In the study by Kulik *et al*,^15^ all YP undergoing cataract surgery with primary intraocular lens implantation developed flare-ups afterwards. Among 34 eyes, 18 eyes (53%) underwent glaucoma surgeries, 15 (44%) underwent YAG laser capsulotomy or equivalent surgical procedures and macular oedema developed in five eyes (15%). Similarly, in the study by Leinonen *et al*,^11^ secondary glaucoma occurred in 19 (73%) YP postoperatively, synechiae in 8 (31%) and macular oedema in 3 (12%). Thus, in this selected population with cataract, concomitant complications often occurred, even long after disease onset. The impact of systemic treatment on these subsequent complications was not addressed other than acute management with intraocular or systemic glucocorticoids.

### Meta-analysis results

**Figure 2:**
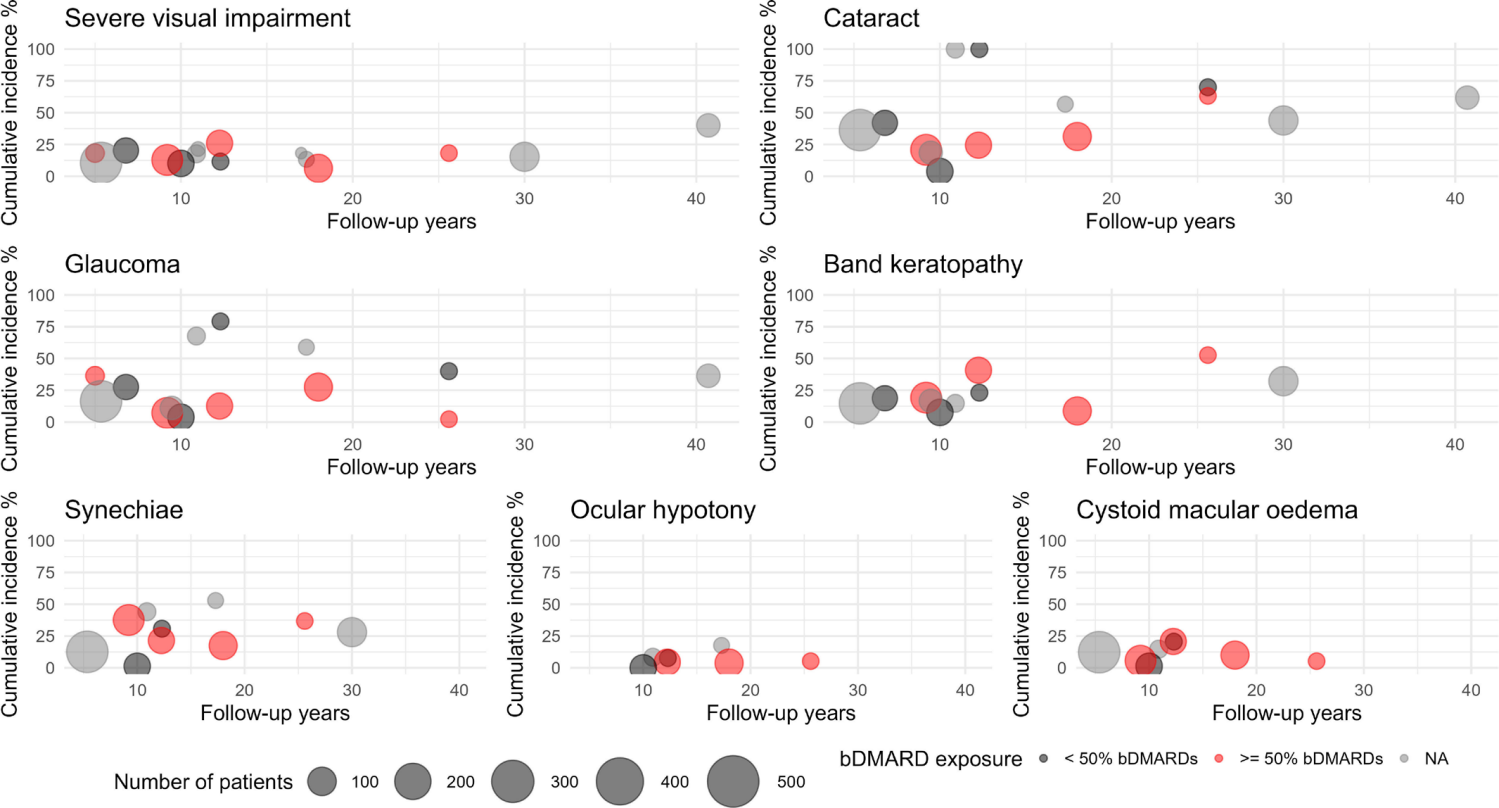
Bubble plot displaying distribution of proportions of each outcome across studies by number of reported median follow-up years (x axis) and number of cases (bubble diameter). bDMARDs, biological disease-modifying antirheumatic drugs. NA: not applicable.

At the last follow-up time point, the proportion of YP with active uveitis in the examined cohorts was high, ranging from a minimum of 15% at 25 years^16^ to a maximum of 52% at 25 years^17^ (figure 2). Cystoid macular oedema was recorded in 11 studies with incidence estimates ranging from 3.7%^12^ at 18 years in a cohort of YP receiving bDMARDs in 54.2% of cases to 17.6% at 17 years in a cohort of children and YP receiving biologics only in 30% of cases^18^ (figure 2). The estimated yearly incidence of this severe manifestation has been reported as 0.01/eye-years (EY).^13^

Severe visual impairment was recorded in a minimum of 6% of YP after 18 years of follow-up in a population-based Nordic study,^12^ up to a maximum of 40% after 40 years of follow-up in a cohort of YP diagnosed between 1944 and 1981 in Finland, excluding deaths (figure 2).^19^ The pooled estimate was 14.2% at 18 years (table 3). In the study by Cann and colleagues on a population of JIA and non-JIA uveitis, the incidence rate of severe visual impairment was 0.01/EY.^13^

**Table 3:** Pooled estimates and meta-analytic parameters for each outcome, considering all studies based on a random effects model on cumulative incidence (proportions)

Complication	Contributing studies (n)	Mean years of follow-up (SD)	Pooled estimate % (95% CI)	I^2^	Moderation test p value for follow-up years	Moderation test p value for year of publication	Egger’s test p value
Severe visual impairment	18	13.5 (10)	14.2 (9.9 to 19.2)	62.7%	0.09	0.14	0.01
Cataract	17	13.3 (10.4)	44.3 (27.5 to 61.7)	97.7%	0.57	0.24	0.01
Glaucoma	16	11.2 (9.1)	27.3 (16.7 to 39.4)	95.2%	0.76	0.58	0.16
Band keratopathy	13	11.4 (8.2)	18.6 (12.4 to 25.6)	79.4%	0.02	0.02	0.34
Posterior synechiae	11	12.5 (8.1)	23.2 (14 to 34)	91.9%	0.30	0.16	0.19
Ocular hypotony	8	14.3 (4.3)	3.3 (0.6 to 7.9)	72.1%	0.91	0.04	0.07
Cystoid macular oedema	11	9.8 (4.9)	7.7 (3.9 to 12.6)	81.8%	0.57	0.02	0.13

For each complication, the number of studies contributing to the value and the weighted mean disease duration are also shown. Heterogeneity is reported by the I2 (%) estimate, and the risk of reporting bias is shown as the Egger’s test p value. The moderation effect (p value of the moderation test) is reported for years of follow-up and year of publication. Estimates are shown with one decimal and p values with two decimals for readability.

In table 3, we report the PE of the cumulative incidence of JIA-associated ocular complications in the studies with comparable measurements. These assessments showed that cataract was the most reported complication (pooled estimate 44.3% (95% CI 27.5% to 61.7%)), followed by glaucoma and band keratopathy.

**Figure 3:**
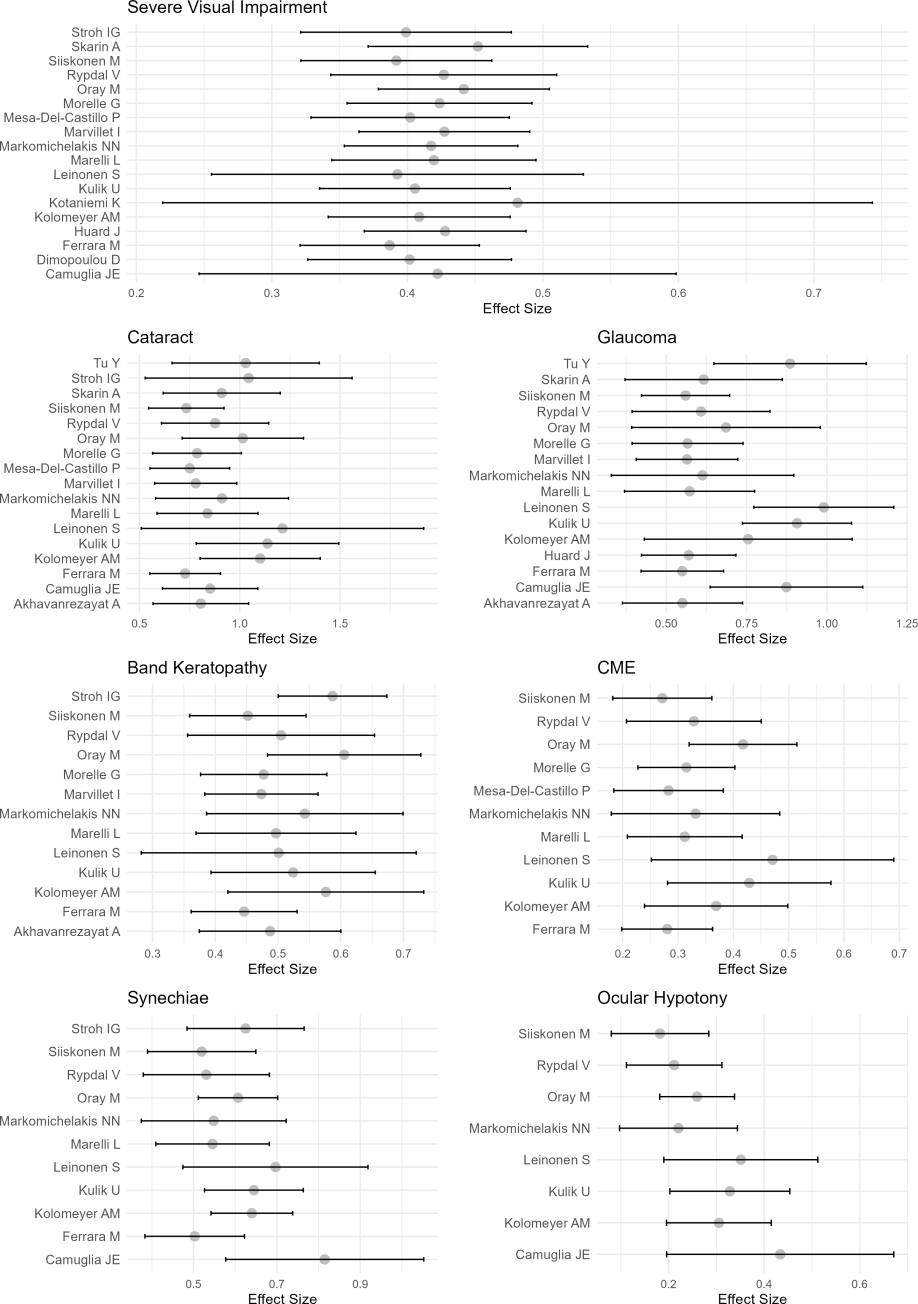
Forest plot of effect sizes of each contributing study for each outcome (sensitivity analysis). CME, cystoid macular oedema.

Significant heterogeneity was found for all outcomes (table 3). We observed a significant effect of year of publication and number of follow-up years for band keratopathy and ocular hypotony, but it was not confirmed for other outcomes (table 3). Since outcomes were discussed in very small studies on surgical YP, inflated effect sizes may potentially explain heterogeneity (figure 3). Egger’s test for risk of reporting bias showed significant asymmetry for severe visual impairment and cataract (table 3 and online supplemental material). The leave-one-out test highlighted that the study from Huard *et al*, reporting on the use of adalimumab, significantly impacted the heterogeneity of the estimate on severe visual impairment, suggesting that the use of biologics, especially in a step-up approach, may significantly decrease the risk of developing serious manifestations and complications. Even though the Egger’s test yielded p values <0.01 for cataract, no single study significantly impacted the final estimate.

Nevertheless, consistent results across studies for all outcomes, as leave-one-out p values were below 0.001 for glaucoma, band keratopathy, posterior synechiae, hypotony and cystoid macular oedema (test results shown in online supplemental material).

We observed that in the study by Marvillet *et al* published before TNFi were licensed for JIA and uveitis, 29/69 JIAU patients presented complications at baseline.^9^ At the last follow-up (median 6 years), the number of YP with complications was 42/69, but only 50% of cases had been treated with csDMARDs and 13/69 (19%) with biologics. Similarly, in the study by Oray *et al*,^19^ YP had a high number of complications and surgeries at baseline (up to 75%). In this study, 68% of subjects were treated with bDMARDs and at the final visit, the number of YP with impaired vision or legal blindness had decreased slightly (33% vs 28% and 17% vs 15%, respectively).

We estimated the cumulative percentages of severe visual impairment, cataract, glaucoma, band keratopathy, posterior synechiae, ocular hypotony and cystoid macular oedema in two groups of studies—those with low (<50%) and high (≥50%) exposure to biologics (table 4). The moderation effect of biologics exposure was not statistically significant in any of the outcomes considered. However, the PE for the cumulative incidence of severe visual impairment, cataract, glaucoma and cystoid macular oedema were numerically lower in the high-exposure group. Conversely, the PE for band keratopathy, posterior synechiae and ocular hypotony were higher in the low exposure group. Importantly, in the study by Huard *et al*, where all YP received weekly adalimumab, most complications were present prior to treatment initiation. In contrast, in the study by Marelli *et al*, where 75% of YP received biologics, complications may have occurred during follow-up, possibly between therapeutic steps. Nevertheless, both studies notably reported a low number of complications overall.

**Table 4:** Moderation effect according to bDMARDs exposure

Complications	Contributing studies (n)	PE % (95% CI)	PE (≥50% exposure to bDMARDs)	PE (<50% exposure to bDMARDs)	I^2^	Moderation test p value
Severe visual impairment	11	12.1 (7.3 to 17.9)	11.2 (0–37.9)	15.54 (6.8–27)	85.7	0.49
Cataract	11	37.7 (37.7 to 18.6)	28.7 (0–96.4)	51.04 (19.5–82.1)	98.1	0.34
Glaucoma	11	24.0 (12.2 to 38.3)	15.1 (0–67.3)	35.8 (15.8–58.9)	94.6	0.15
Band keratopathy	9	18.5 (10.3 to 28.5)	26.8 (2-65)	15.2 (5.4–28.9)	90.1	0.23
Posterior synechiae	7	19.1 (8.9 to 32.1)	27.4 (0–83)	10.0 (0.3–31.0)	91.1	0.17
Ocular hypotony	6	1.8 (0.1 to 5.4)	4.3 (0–23.5)	0.7 (0.0–4.5)	72.6	0.14
Cystoid macular oedema	9	6.7 (2.8 to 12.2)	6.7 (0–40.5)	8.3 (1.1–21.5)	88.5	0.81

bDMARDs, biologic disease-modifying antirheumatic drugs; n, number of studies; PE, pooled estimate.

## Discussion

Our systematic review and meta-analysis identified 22 heterogeneous studies published in the 21st-century that reported data on complications of JIAU in long-term uveitis or adulthood. Most studies were small and retrospective, with overall suboptimal quality, as only 10/22 were rated as good. Heterogeneity was high for all outcomes and only partially influenced by differences in follow-up duration and publication year. Other contributing factors may include small sample sizes, the setting of YP enrolment (JIA-specific screening clinics versus ophthalmologic reference centres for uveitis versus ophthalmologic reference centres for surgical procedures), when outcome measures are assessed and how each outcome is defined by each author. Our studies encompass a broad period of time wherein management strategies, as well as knowledge and awareness about JIAU complications, have seen a dramatic evolution. Despite a recent expert consensus for the elaboration of international recommendations in some areas,^5 7^ there remain some disagreements that could potentially influence outcomes and should be addressed by future ad hoc studies.^20^

In our review, the pooled estimate of the cumulative incidence of severe visual impairment in at least one eye was comparable to the 9.2% reported in an earlier systematic literature review.^6^ However, it bears noting that this complication appeared to be numerically less frequent in cohorts with higher exposure to biologic therapies, and PE consistently trended lower in these populations across most outcomes, in particular cataract and glaucoma—the most common complications in JIAU. In the aforementioned systematic literature review, cataract was estimated to complicate 20.5% of cases, 18.9% of glaucoma and 15.7% of band keratopathy. Our PE in cohorts with low biologics exposure were much higher for cataract and glaucoma (whereas they were significantly improved in cohorts with higher bDMARDs exposure).

Population-based studies and registries from Northern Europe and Spain tended to report fewer complications. By contrast, series from tertiary ophthalmology clinics may have been hampered by referral bias, with more complicated cases. For this reason, we applied the leave-one-out method for bias detection, suspecting that surgical-only studies may negatively significantly impact our summary. However, Huard *et al* was the sole study that reported a statistically significant effect with weekly adalimumab after the implementation of a strict and aggressive protocol,^21^ thus confirming that aggressive treatment may improve outcomes.

Another possible source of bias may be time to treatment, which was variably reported in the studies included in our review. A key limitation of our study was the difficulty in distinguishing between complications present at baseline and those that developed during follow-up. This is particularly relevant because delayed diagnosis and late referrals can inflate complication rates. Indeed, Huard *et al*^21^ reported that most YP already had ocular damage at baseline. In the study by Oray *et al,*^19^ wherein baseline was considered as the first referral to a tertiary centre with a mean disease duration of more than 12 years, complication rates at baseline affected 67% of eyes. Although the proportion of patients with severe visual impairment decreased from 17% at referral to 15% at the final visit, there was no temporal treatment specification during follow-up, and the total number of eyes with complications increased to 72%. Thus, the true protective effect of biologics against new complications may likely be underestimated. It is hypothesised that in the studies reporting a high percentage of biologics exposure, these effective treatments may have been applied years after disease onset, as suggested by Oray *et al* and Rypdal *et al*, wherein bDMARDs were introduced in 22% of patients within 8 years of onset and 54% within 18 years of onset, respectively.^12^ Standardised reporting to distinguish baseline from incident ocular damage will be essential in future studies. Additionally, longer delays before initiating bDMARDs for anterior uveitis—whether JIAU or idiopathic—are consistently associated with higher risks of vision loss, cataract and glaucoma,^22^,^24^ reinforcing the importance of early biologic treatment. This appears to challenge the current recommendations of a first trial with a course of methotrexate, and only subsequently the introduction of adalimumab.^4^

Across all settings, a subset of JIAU YP emerged with refractory relapsing disease requiring long-term monitoring for complications well into adulthood. In the study by Skarin *et al*, 40% of individuals with an active follow-up over 20 years after disease onset were displaying signs of active JIAU. Adalimumab is an extremely effective biologic therapy for JIAU, as demonstrated by several randomised controlled trials.^25^,^29^ However, Rypdal *et al* reported that even though patients were treated with bDMARDs at some point during follow-up in 80% of cases, 20 YP still developed complications within 8 years, including seven previously unaffected patients.^12^ Additionally, one recent real-life study showed a 5.2% rate of vision loss over 5 years in YP with uveitis, alongside increased risk for cataract and synechiae, despite systematic treatment with adalimumab.^22^

Dose escalation during flares is effective in approximately 75% of children and YP. However, a persistent concern remains that some patients may develop antibodies against adalimumab,^30^ and a significant subgroup of non-responders carries a substantial risk of complications even with dose adjustment.^31^ Although this subset of patients may benefit most from additional therapeutic options, there is little consensus to date regarding their optimal management with existing strategies.^20^

Comparisons with idiopathic chronic anterior uveitis yielded conflicting results. Some reports suggested higher risks of ocular hypertension and glaucoma but lower risks of macular oedema in JIAU^13 32^ (whereas others found a greater likelihood of severe vision loss for JIAU).^14^ Conversely, registry data from Spain indicated lower complication rates in JIAU, which may be attributable to the earlier and broader use of csDMARDs and biologics.^33^ These trends are consistent with the effect of systematic screening programmes and the aggressive treat-to-target approach currently recommended for JIAU.^4 34^

Therapeutic trials for JIAU remain limited in both duration and scope, as chronic anterior uveitis still needs more therapeutic options and more standardised management. No trial has extended beyond 2 years or into adulthood,^35 36^ but this would be very meaningful for investigating therapeutic tapering, if not withdrawal, treatment failures and the long-term impact of these treatments in terms of safety and damage. Recent initiatives, such as the ADJUST trial of adalimumab withdrawal^37^ and long-term baricitinib studies^38^ (NCT04200833), aimed to address these gaps. Among second-line options, tocilizumab and infliximab remain the most widely supported,^5^ with abatacept,^39^ JAK inhibitors and rituximab as possible alternatives in refractory disease. While the controlled trial of baricitinib was negative, case series suggest potential benefit in selected individuals,^40 41^ and the JAHW trial of baricitinib and adalimumab is ongoing. Encouraging preliminary results have also emerged with upadacitinib.^40 42^ IL-1 inhibitors have shown little efficacy in JIAU,^43^ but IL-17 blockade warrants further exploration.^44^

The question of treatment withdrawal remains unresolved. The American College of Rheumatology recommends considering tapering after 2 years of disease quiescence or remission, but the ADJUST trial demonstrated high relapse rates after adalimumab discontinuation.^37^ In the latter, the main limitation was that adalimumab withdrawal was abrupt and possible after only 12 months of disease control, whereas in clinical practice, spacing is usually tried first. Indeed, lowering to the least effective dose might be a sustainable approach versus complete discontinuation. Unfortunately, few observational studies reported treatment discontinuation rates, thus limiting our ability to quantify the associated risk of relapse.

When complications arise, surgical management remains limited to cataract surgery or capsulotomy for glaucoma and synechiae. These procedures are rarely definitive and often complicated by postoperative inflammation. Optimising surgical timing and improving perioperative management may result in better outcomes.

Overall, our review underscores the persistent burden of JIAU, with complications accruing even 15 years after disease onset, echoing findings from 20th-century cohorts. Nevertheless, our subgroup analysis comparing high versus low biologic exposure suggests that early and aggressive biologic treatment may significantly improve outcomes in patients with JIAU. Future studies should assess long-term outcomes in larger, comparable cohorts, using harmonised outcome definitions and a clearer temporal separation. Given the similarities between JIAU and paediatric-onset idiopathic chronic anterior uveitis—two rare diseases—future studies including both subsets of patients may help overcome the limitation of small sample sizes. Ongoing initiatives prioritising YP well-being, equity and inclusion^45^,^47^ are encouraging steps in this direction. In parallel, preclinical efforts, such as the Childhood Ocular Inflammatory Disease Biobank in London, will be key to uncovering disease mechanisms and identifying novel therapeutic targets.

In conclusion, JIAU remains a common and often persistent complication of JIA, associated with debilitating long-term visual outcomes that significantly affect quality of life.^48^ Although biologic therapies—especially adalimumab—have yielded encouraging results to date, complications remain frequent in non-responders and in countries where access to biologics is limited. Early diagnosis, as well as timely and aggressive treatment, remains essential, and future research should aim to refine treatment targets and optimise outcomes across diverse populations.

## Supplementary material

10.1136/rmdopen-2025-006071 online supplemental file 1

## Data Availability

Data are available upon reasonable request.
