# Supplementary material for: Long-term outcomes of JIA-associated uveitis: a systematic review and meta-analysis
Source: RMD Open. 2025 Nov 27;11(4):e006071. doi: 10.1136/rmdopen-2025-006071 (PMC12666193; doi:10.1136/rmdopen-2025-006071)

**Supplementary Material**

1. Supplementary Data S1: Pubmed search string
2. Supplementary Figure S2: Forest plots for individual outcomes - Heterogeneity
3. Supplementary Table S3: Table of leave-one-out results for sensitivity
4. Supplementary Figure S4: Funnel plots for individual outcomes - risk of reporting bias
5. Supplementary Data S1: **Pubmed search string**

- damage in general: ((("arthritis, juvenile"[MeSH Terms] OR ("arthritis"[All Fields] AND "juvenile"[All Fields]) OR "juvenile arthritis"[All Fields] OR ("juvenile"[All Fields] AND "idiopathic"[All Fields] AND "arthritis"[All Fields]) OR "juvenile idiopathic arthritis"[All Fields]) NOT ("systemic"[All Fields] OR "systemically"[All Fields] OR "systemics"[All Fields])) AND ("uveitis"[MeSH Terms] OR "uveitis"[All Fields] OR "uveitides"[All Fields]) AND ("damage"[All Fields] OR "damaged"[All Fields] OR "damages"[All Fields] OR "damaging"[All Fields])) NOT ("review"[Publication Type] OR "review literature as topic"[MeSH Terms] OR "review"[All Fields])
- cataract: ((("arthritis, juvenile"[MeSH Terms] OR ("arthritis"[All Fields] AND "juvenile"[All Fields]) OR "juvenile arthritis"[All Fields] OR ("juvenile"[All Fields] AND "idiopathic"[All Fields] AND "arthritis"[All Fields]) OR "juvenile idiopathic arthritis"[All Fields] OR ("systemic"[All Fields] OR "systemically"[All Fields] OR "systemics"[All Fields])) AND ("uveitis"[MeSH Terms] OR "uveitis"[All Fields] OR "uveitides"[All Fields])) NOT ("review"[Publication Type] OR "review literature as topic"[MeSH Terms] OR "review"[All Fields])) AND ("cataract"[MeSH Terms] OR "cataract"[All Fields] OR "cataracts"[All Fields] OR "cataractic"[All Fields] OR "cataractous"[All Fields])
- band keratopathy: ((("arthritis, juvenile"[MeSH Terms] OR ("arthritis"[All Fields] AND "juvenile"[All Fields]) OR "juvenile arthritis"[All Fields] OR ("juvenile"[All Fields] AND "idiopathic"[All Fields] AND "arthritis"[All Fields]) OR "juvenile idiopathic arthritis"[All Fields] OR ("systemic"[All Fields] OR "systemically"[All Fields] OR "systemics"[All Fields])) AND ("uveitis"[MeSH Terms] OR "uveitis"[All Fields] OR "uveitides"[All Fields])) NOT ("review"[Publication Type] OR "review literature as topic"[MeSH Terms] OR "review"[All Fields])) AND (("band"[Journal] OR "band"[All Fields]) AND ("keratopathies"[All Fields] OR "keratopathy"[All Fields]))
- ((("arthritis, juvenile"[MeSH Terms] OR ("arthritis"[All Fields] AND "juvenile"[All Fields]) OR "juvenile arthritis"[All Fields] OR ("juvenile"[All Fields] AND "idiopathic"[All Fields] AND "arthritis"[All Fields]) OR "juvenile idiopathic arthritis"[All Fields] OR ("systemic"[All Fields] OR "systemically"[All Fields] OR "systemics"[All Fields])) AND ("uveitis"[MeSH Terms] OR "uveitis"[All Fields] OR "uveitides"[All Fields])) NOT ("review"[Publication Type] OR "review literature as topic"[MeSH Terms] OR "review"[All Fields])) AND (("posterior"[All Fields] OR "posteriors"[All Fields]) AND ("synechia"[All Fields] OR "synechiae"[All Fields] OR "synechiaes"[All Fields] OR "synechias"[All Fields]))
- ((("arthritis, juvenile"[MeSH Terms] OR ("arthritis"[All Fields] AND "juvenile"[All Fields]) OR "juvenile arthritis"[All Fields] OR ("juvenile"[All Fields] AND "idiopathic"[All Fields] AND "arthritis"[All Fields]) OR "juvenile idiopathic arthritis"[All Fields] OR ("systemic"[All Fields] OR "systemically"[All Fields] OR "systemics"[All Fields])) AND ("uveitis"[MeSH Terms] OR "uveitis"[All Fields] OR "uveitides"[All Fields])) NOT ("review"[Publication Type] OR "review literature as topic"[MeSH Terms] OR "review"[All Fields])) AND (("visual"[All Fields] OR "visualisation"[All Fields] OR "visualisations"[All Fields] OR "visualise"[All Fields] OR "visualised"[All Fields] OR "visualises"[All Fields] OR "visualising"[All Fields] OR "visualization"[All Fields] OR "visualizations"[All Fields] OR "visualize"[All Fields] OR "visualized"[All Fields] OR "visualizer"[All Fields] OR "visualizers"[All Fields] OR "visualizes"[All Fields] OR "visualizing"[All Fields] OR "visually"[All Fields] OR "visuals"[All Fields]) AND "loss"[All Fields])

1. Supplementary Figure S2: **Forest plots for individual outcomes - Heterogeneity**

**
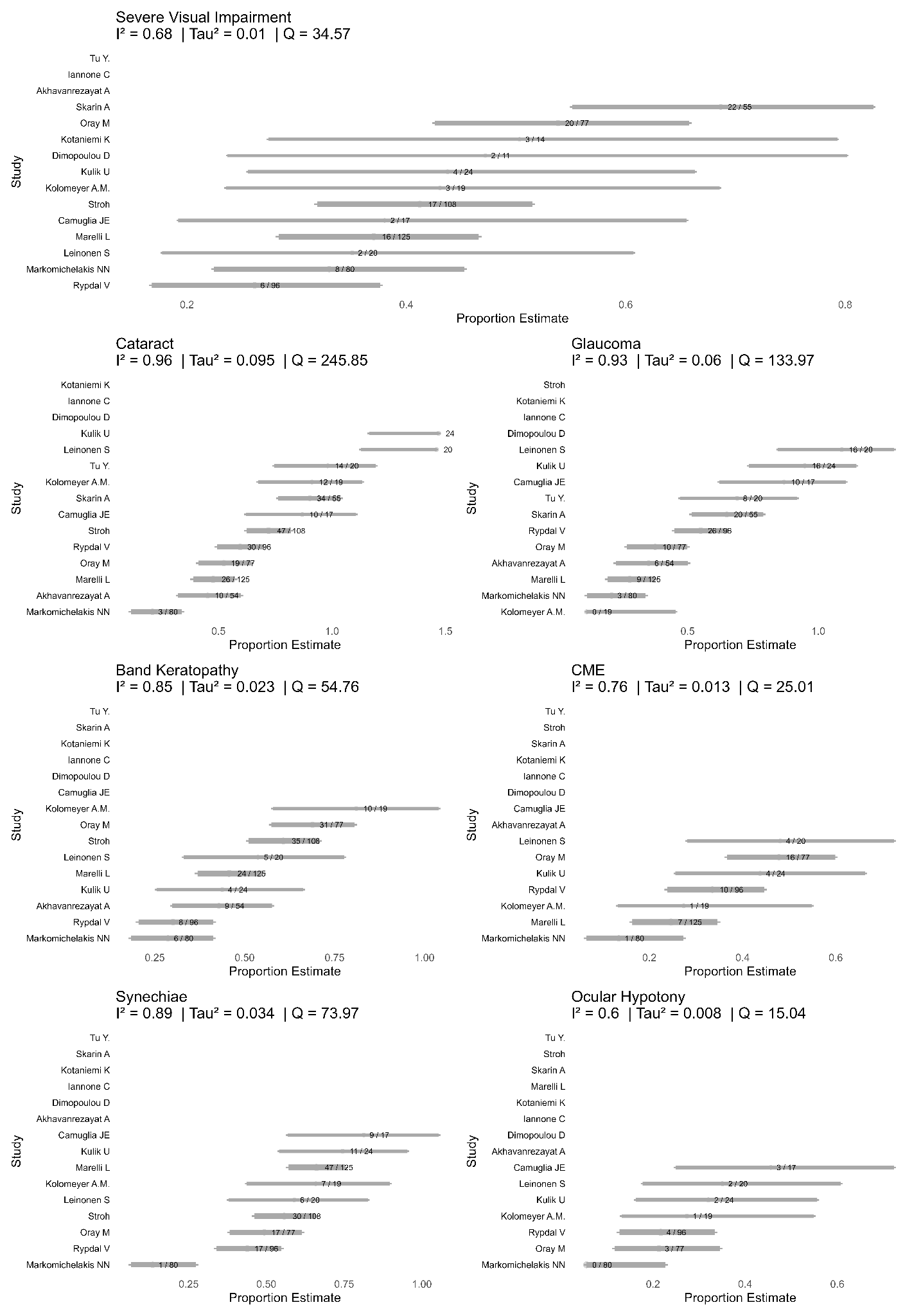
**

1. Supplementary Table S3: **Table of leave one out results for sensitivity** (estimate, p-values, I^2^, Tau^2^, and H^2^ for each contributing study)

| Outcome | Study ID | Estimate | P value | I^2^ | Tau^2^ | H^2^ |
| --- | --- | --- | --- | --- | --- | --- |
| Severe visual impairment | 1 | 0.39 | <0.00 | 45.41 | 0.00 | 1.83 |
|  | 2 | 0.41 | <0.00 | 66.86 | 0.01 | 3.02 |
|  | 3 | 0.42 | <0.00 | 71.60 | 0.01 | 3.52 |
|  | 4 | 0.42 | <0.00 | 71.51 | 0.01 | 3.51 |
|  | 5 | 0.44 | <0.00 | 60.72 | 0.01 | 2.55 |
|  | 6 | 0.42 | <0.00 | 71.71 | 0.01 | 3.53 |
|  | 7 | 0.42 | <0.00 | 71.37 | 0.01 | 3.49 |
|  | 8 | 0.42 | <0.00 | 71.39 | 0.01 | 3.49 |
|  | 9 | 0.43 | <0.00 | 68.35 | 0.01 | 3.16 |
|  | 11 | 0.42 | <0.00 | 71.67 | 0.01 | 3.53 |
|  | 12 | 0.42 | <0.00 | 69.56 | 0.01 | 3.28 |
|  | 15 | 0.43 | <0.00 | 68.38 | 0.01 | 3.16 |
| Cataract | 1 | 0.80 | <0.00 | 97.66 | 0.19 | 42.66 |
|  | 2 | 0.83 | <0.00 | 97.47 | 0.18 | 39.47 |
|  | 3 | 0.80 | <0.00 | 97.81 | 0.19 | 45.57 |
|  | 5 | 0.83 | <0.00 | 97.45 | 0.18 | 39.20 |
|  | 6 | 0.74 | <0.00 | 96.69 | 0.12 | 30.25 |
|  | 7 | 0.74 | <0.00 | 96.76 | 0.13 | 30.90 |
|  | 9 | 0.86 | <0.00 | 96.90 | 0.15 | 32.25 |
|  | 10 | 0.84 | <0.00 | 97.49 | 0.17 | 39.84 |
|  | 11 | 0.80 | <0.00 | 97.79 | 0.19 | 45.16 |
|  | 12 | 0.81 | <0.00 | 97.46 | 0.19 | 39.39 |
|  | 14 | 0.79 | <0.00 | 97.75 | 0.18 | 44.54 |
|  | 15 | 0.84 | <0.00 | 97.23 | 0.18 | 36.12 |
| Glaucoma | 1 | 0.54 | <0.00 | 95.41 | 0.10 | 21.80 |
|  | 2 | 0.57 | <0.00 | 95.08 | 0.10 | 20.33 |
|  | 3 | 0.52 | <0.00 | 95.24 | 0.09 | 21.03 |
|  | 5 | 0.55 | <0.00 | 95.12 | 0.10 | 20.50 |
|  | 6 | 0.51 | <0.00 | 94.67 | 0.08 | 18.77 |
|  | 7 | 0.50 | <0.00 | 93.73 | 0.07 | 15.95 |
|  | 9 | 0.59 | <0.00 | 94.40 | 0.09 | 17.86 |
|  | 10 | 0.57 | <0.00 | 95.24 | 0.10 | 21.01 |
|  | 11 | 0.59 | <0.00 | 94.97 | 0.09 | 19.87 |
|  | 14 | 0.54 | <0.00 | 95.66 | 0.10 | 23.06 |
|  | 15 | 0.58 | <0.00 | 94.36 | 0.09 | 17.72 |
| Band keratopathy | 2 | 0.46 | <0.00 | 83.35 | 0.02 | 6.01 |
|  | 5 | 0.51 | <0.00 | 84.32 | 0.02 | 6.38 |
|  | 6 | 0.50 | <0.00 | 88.40 | 0.03 | 8.62 |
|  | 7 | 0.49 | <0.00 | 88.73 | 0.03 | 8.87 |
|  | 9 | 0.52 | <0.00 | 83.89 | 0.02 | 6.21 |
|  | 10 | 0.50 | <0.00 | 88.07 | 0.03 | 8.38 |
|  | 11 | 0.46 | <0.00 | 83.71 | 0.02 | 6.14 |
|  | 12 | 0.47 | <0.00 | 86.02 | 0.03 | 7.16 |
|  | 15 | 0.49 | <0.00 | 86.91 | 0.03 | 7.64 |
| CME | 2 | 0.28 | <0.00 | 66.60 | 0.01 | 2.99 |
|  | 5 | 0.31 | <0.00 | 78.86 | 0.02 | 4.73 |
|  | 6 | 0.30 | <0.00 | 80.42 | 0.02 | 5.11 |
|  | 7 | 0.29 | <0.00 | 78.25 | 0.01 | 4.60 |
|  | 9 | 0.35 | <0.00 | 60.33 | 0.01 | 2.52 |
|  | 11 | 0.32 | <0.00 | 80.93 | 0.02 | 5.24 |
|  | 15 | 0.33 | <0.00 | 76.34 | 0.02 | 4.23 |
| Synechiae | 2 | 0.56 | <0.00 | 90.66 | 0.04 | 10.71 |
|  | 3 | 0.52 | <0.00 | 89.81 | 0.03 | 9.82 |
|  | 5 | 0.56 | <0.00 | 90.01 | 0.04 | 10.01 |
|  | 6 | 0.53 | <0.00 | 90.63 | 0.04 | 10.67 |
|  | 7 | 0.54 | <0.00 | 91.57 | 0.04 | 11.86 |
|  | 9 | 0.59 | <0.00 | 67.12 | 0.01 | 3.04 |
|  | 11 | 0.54 | <0.00 | 91.33 | 0.04 | 11.54 |
|  | 12 | 0.55 | <0.00 | 90.23 | 0.04 | 10.24 |
|  | 15 | 0.53 | <0.00 | 89.33 | 0.04 | 9.37 |
| Ocular  hypotony | 2 | 0.22 | <0.00 | 69.42 | 0.02 | 3.27 |
|  | 3 | 0.18 | <0.00 | 60.56 | 0.01 | 2.54 |
|  | 5 | 0.22 | <0.00 | 68.42 | 0.02 | 3.17 |
|  | 6 | 0.20 | <0.00 | 69.87 | 0.01 | 3.32 |
|  | 7 | 0.20 | <0.00 | 70.75 | 0.01 | 3.42 |
|  | 9 | 0.24 | <0.00 | 0.00 | 0.00 | 1.00 |
|  | 11 | 0.21 | <0.00 | 72.10 | 0.01 | 3.58 |

1. Supplementary Figure S4: **Funnel plots for individual outcomes - risk of reporting bias**


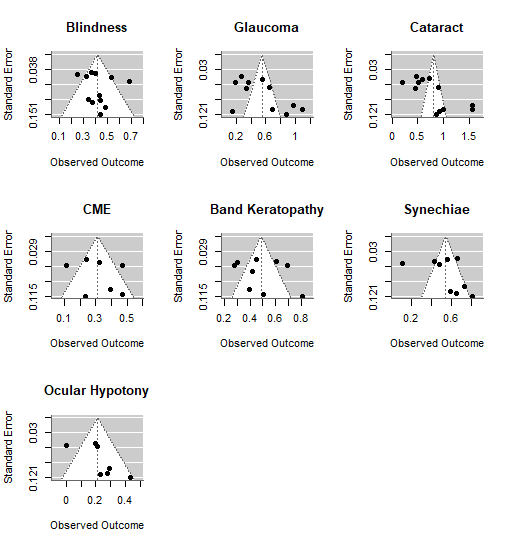

Supplement: online supplemental file 1 [file rmdopen-11-4-s001.docx]
